# Skin Pigmentation Influence on Pulse Oximetry Accuracy: A Systematic Review and Bibliometric Analysis

**DOI:** 10.3390/s22093402

**Published:** 2022-04-29

**Authors:** Ana M. Cabanas, Macarena Fuentes-Guajardo, Katina Latorre, Dayneri León, Pilar Martín-Escudero

**Affiliations:** 1 Departamento de Física, Universidad de Tarapacá, Arica 1010069, Chile; 2 Departamento de Tecnología Médica, Universidad de Tarapacá, Arica 1010069, Chile; mafuentesg@academicos.uta.cl (M.F.-G.); kalatorrep@academicos.uta.cl (K.L.); 3 Departamento de Educación Física, Universidad de Tarapacá, Arica 1010069, Chile; dleon@academicos.uta.cl; 4 Medical School of Sport Medicine, Faculty of Medicine, Universidad Complutense de Madrid, 28040 Madrid, Spain; pmartinescudero@med.ucm.es

**Keywords:** pulse oximetry, oxygen saturation, skin pigmentation, accuracy, photoplethysmography

## Abstract

Nowadays, pulse oximetry has become the standard in primary and intensive care units, especially as a triage tool during the current COVID-19 pandemic. Hence, a deeper understanding of the measurement errors that can affect precise readings is a key element in clinical decision-making. Several factors may influence the accuracy of pulse oximetry, such as skin color, body temperature, altitude, or patient movement. The skin pigmentation effect on pulse oximetry accuracy has long been studied reporting some contradictory conclusions. Recent studies have shown a positive bias in oxygen saturation measurements in patients with darkly pigmented skin, particularly under low saturation conditions. This review aims to study the literature that assesses the influence of skin pigmentation on the accuracy of these devices. We employed the Preferred Reporting Items for Systematic Reviews and Meta-Analyses (PRISMA) statement to conduct a systematic review retrospectively since February 2022 using WOS, PubMed, and Scopus databases. We found 99 unique references, of which only 41 satisfied the established inclusion criteria. A bibliometric and scientometrics approach was performed to examine the outcomes of an exhaustive survey of the thematic content and trending topics.

## 1. Introduction

New non-invasive methods that can be applied in laboratory tests for patients or healthy adults as athletes for accurate monitoring of health variables have been developed in recent years. These methods include heart rate determination by electrocardiography, photoplethysmography (PPG) [1,2], light spectroscopy [3], and pulse oximetry [4,5,6]. PPG is an optical, simple, inexpensive, and non-invasive method for detecting arterial pulsation for measuring changes in blood volume according to the amount of incident light reflected or transmitted [7,8].

Pulse oximeter devices incorporating PPG are employed to evaluate peripheral blood oxygen saturation based on the measurement of red and infrared light absorption levels, directly influenced by hemoglobin levels [4,5]. The arterial oxygen saturation ($SaO_{2}$) represents the percentage of binding sites on hemoglobin carrying oxygen. As the partial pressure of oxygen ($pO_{2}$) rises, more $O_{2}$ molecules are available to bind with hemoglobin [9,10]. In clinical practice, oxygen saturation by pulse oximetry ($S_{p}O_{2}$) gives information about the amount of oxygen available in the tissues by measuring how many of these binding sites are mixed with oxygen.

During exercise stress tests or respiratory rehabilitation therapies, pulse oximetry is a valuable method to determine the limit of cardiopulmonary stress, which is characterized by a relevant decrease in the value of oxygen saturation [11]. This technique has become the standard in primary and intensive care units and other clinical settings, such as anesthesiology, which requires constant and frequent monitoring of vital signs [1]. Besides, pulse oximetry has been widely used in rehabilitated COVID-19 patients with risks of developing respiratory distress [12,13,14,15,16,17,18]. Indeed, a recent retrospective cohort study of high-risk patients with COVID-19 pneumonia found that mortality was 48% lower in patients who used a pulse oximeter to monitor $SpO_{2}$ than those without one [19]. Therefore, the current World Health Organization COVID-19 management guideline recommends that symptomatic people with COVID-19 to use a home pulse oximeter for self $S_{p}O_{2}$ monitoring “as part of a package of care” [20].

Traditional pulse oximeters are composed of two principal components: a light-emitting diode (LED) with two or more wavelengths and a photodetector [21]. The penetration depth of the light is determined by the wavelength and the distance between the light source and the photodetector. Long wavelengths such as red (660–700 nm) and infrared light, are suitable for measuring deep-tissue blood flow [22]. Conventional red and infrared pulse oximeters are reliable at rest and in normoxic or hyperoxic conditions, but have presented worsening accuracy in hypoxic conditions [23]. Different factors have been examined to determine the influence on the accuracy of pulse oximetry such as skin pigmentation [24], body temperature [25], altitude [26], barometric pressure [27], nail polish or henna [28], or motion artifact [29]. Under intense exertion, motion artifacts complicate the use of the pulse oximeter and the accurate interpretation of the data [29,30,31], especially with heart rate higher than 150 beats per minute [32]. Regarding skin pigmentation, several studies have also reported inaccurate readings in $S_{p}O_{2}$ and heart-rate measurements by PPG in dark-skinned subjects because of the interference of melanin with the quality of the reflected signal [33,34,35,36,37].

Using multi-wavelength devices improves accuracy because they are less sensitive than the classical devices to the variations in the hemoglobin saturation [38]. Analyses of PPG signals at different wavelengths have shown that not only the accuracy of $S_{p}O_{2}$ is improved. However, it is possible to extract more information about skin pathologies at various tissue depths because other substances in the blood (such as methemoglobin and carboxyhemoglobin) can be detected [39]. Green-wavelength pulse oximeters display the greatest modulation depth with pulsatile blood absorption, i.e., a greater absorbance for both deoxyhemoglobin and oxyhemoglobin than infrared light [40].

Skin pigmentation impact on the accuracy of $S_{p}O_{2}$ monitoring has long been studied [24,41,42,43,44,45,46,47,48,49,50,51]. One of the earliest studies on this topic in 1976 already reported reading errors in dark-skinned patients, reflecting lower blood oxygen saturation values [41]. More recent studies have also revealed that dark skin pigmentation influences the accuracy and performance of pulse oximeters devices, resulting in overestimations [17,49,50,51,52,53] with increased incidence in the risk for occult hypoxemia ($SO_{2}<88\%$ despite normal $SpO_{2}>92\%$) [54,55]. This bias is a matter of major concern since drops of only 2% are of particular importance in respiratory rehabilitation, studies of sleep apnea, and athletes performing physical efforts because they can lead to severe causes for the patient, requiring an external oxygen supply or even hospitalization.

Conversely, some older studies did not identify a considerable bias related to skin pigmentation [44,45,46,56] suggesting that dark skin pigmentation does not affect the quality of the signal [47]. However, these findings may be misleading because of a small sample size of the dark-skinned population or a weak correlation with skin pigmentation by using uncertain descriptors of ancestry to classify the population.

Due to this increasing evidence, several health organizations and healthcare professionals have raised growing concerns about the clinical accuracy of pulse oximetry in dark-skinned patients [20,57,58,59,60,61], particularly in detecting occult hypoxemia [54,55]. A recent comprehensive review of different studies comparing $SpO_{2}$ measurements with arterial blood gas analysis $SaO_{2}$ in different patients and conditions has reported accurate but imprecise measurements across all levels of skin pigmentation [61]. Although not all the reported studies assess specifically the influence of skin pigmentation, low-certainty evidence about overestimations in dark-skinned patients was reported. This systematic review aims to summarize the available literature in the main scientific databases which examine the impact of skin pigmentation on pulse oximetry, unraveling the following research questions:

- RQ1: What are the most significant publications and the ongoing research trends for prospect analysis on this topic?
- RQ2: How does skin color affect the accuracy of pulse oximeter devices incorporating photoplethysmography?
- RQ3: On which human populations have studies been conducted to verify these discrepancies and what methods have been employed to classify skin pigmentation?

Section 2 describes the search employed method to identify all relevant references by defining a suitable search query. The inclusion and exclusion criteria are exposed in Section 2.2, and the analysis and data extraction process are presented in Section 2.3. Next, Section 3 introduces the obtained results individually. In Section 4, the corresponding discussion and the four research questions are answered. Finally, the conclusions are presented in Section 4.

## 2. Methodology

We performed a systematic review based on bibliometric analysis to build a fair portrayal of the current research trends on this topic. Systematic reviews provide an exhaustive synthesis of the relevant literature to a specific research question [62,63,64]. The bibliometric analysis permits us to analyze a large amount of information extracted from a scientific database, performing a quantitative evaluation of the research topics [65,66]. Therefore, we have performed a scientometrics study to assess whether there is a lack of accuracy in pulse oximetry devices related to dark skin pigmentation.

### 2.1. Literature Search

We conducted a retrospectively systematic review of electronic databases between January 1975 and February 2022. The Preferred Reporting Items for Systematic Reviews and Meta-Analyses (PRISMA) statement was applied to pre-identified the selected studies indexed in PubMed, Web of Science (WOS), and Scopus databases. The research questions were based on the Patient, Intervention, Comparison, and Outcome (PICO) model, taking into consideration the following aspects: population (patient), pulse oximetry method (intervention), comparison (comparison), and outcomes. Two authors (AMC and MFG) independently performed the bibliographic search, while the search algorithm was reviewed and discussed with the rest of the authors (PM, KL or DL). To construct a suitable query and maximize the search strategy acuteness, the research question included the following keyword and medical subject headings (MESH) combined terms and their related topics:

- “oximetry” according to MeSH terms.
- “pulse oximetry” OR “oximet*” OR “oxygen saturation” to include all the relative references.
- “photoplethysmography” according to MeSH terms.
- “photoplethysmography” OR “PPG” as a generic term that refers to the optical imaging technique for detecting arterial pulsation.
- “skin” OR “pigmentation” OR “racial” OR “race” OR “ethnic*” to include all the relative references.
- “accuracy” OR “precision” OR “error” OR “reliability” OR “bias” to find all relative references.

The final analysis contained primary research about the references that assess skin pigmentation’s impact on pulse oximetry. A literature hand search supplemented these results. We display the full query in Table 1.

### 2.2. Inclusion and Exclusion Criteria

Research based on publications that describe the aforementioned topic was included. Any prospective or retrospective study, including full publications, reviews conference proceedings, technical notes, and reports, were examined. We included studies that explored the accuracy of any type of pulse oximeter regarding skin pigmentation. The level of skin pigmentation was considered by both an objective measurement or scale and ancestry descriptors. If the information of any study was insufficient to fulfill the inclusion criteria, we searched for more information by contacting authors. We excluded the studies where no response was obtained. The exclusion criteria were:

- Non-human focused studies;
- Skin pigmentation influence not evaluated;
- References that do not focus on pulse oximetry.

### 2.3. Data Extraction and Analysis

A suitable query was designed to maximize Scopus, PubMed and WOS results. The five authors performed the quality and eligibility assessment of relevant references. The following information was obtained: author, year of publication, journal, source, citations, author’s keywords, and research outputs. We also analyzed the bibliography of the encountered references to add associated studies on the topic. Titles, abstracts, and author’s keywords were first independently analyzed to get enough information about the eligibility of two authors (AMC and MFG, or PM). Second, we carefully examined the full text to check whether the references satisfy the inclusion criteria by the five authors. The chosen references were ordered by the total received citations from the three databases.

**Figure 1:**
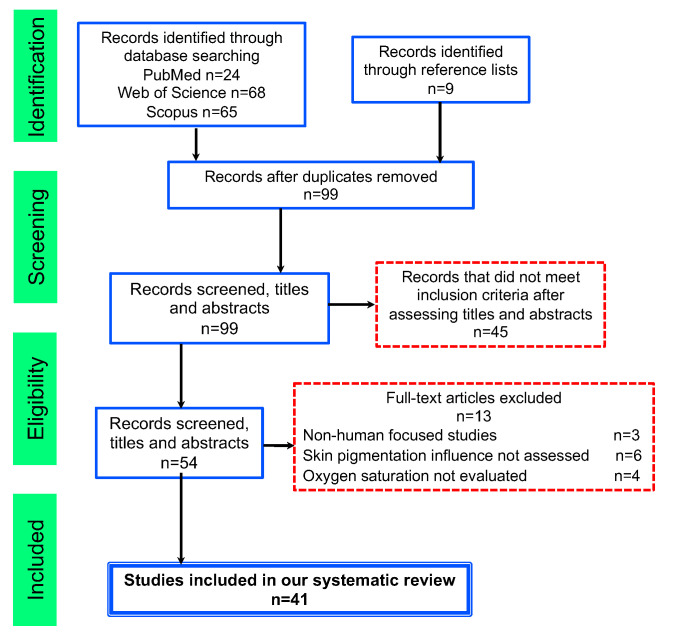
Flowchart consistent with preferred reporting items for systematic reviews (PRISMA) statement.

Figure 1 describes the eligibility screening processes by each stage of the search. After titles and abstract examination, 54 references were potentially appropriate for full-text reading. We excluded references that did not satisfy the inclusion criteria. The principal reasons for exclusion were: studies that did not focus on humans (5); skin pigmentation influence not evaluated (4); oxygen saturation not evaluated (4).

Bibliometrix R-package and Biblioshiny, the webinterface, were employed to unravel the proposed research question [67]. VOSviewer (version 1.6.16) was also used to visualize a knowledge map of the topics and perform a cluster analysis based on keywords and co-occurrence analysis. The co-occurrence technique is usually employed to analyze dynamic patterns and trends of publications related to a specific topic [68].

## 3. Results

We identified 41 studies that fulfilled the inclusion criteria from 31 different sources between 1976 and 2022. We present an overview of this information in Table 2.

Table 3 presents the selected references sorted by year of publication. The first author’s name, publication year, size of the sample, number of dark-skinned subjects, gender, type of participant, and type of oximeter employed are shown.

**Table 3:** Selected studies from the search query sorted by year of publication. First author’s name, publication year, size of the sample, number of dark skinned subjects, gender, type of participant, and type of oximeter employed are shown.

Reference, Year	N	N Dark (%)	Age	Gender (Male/Female)	Type of Participants	Scale or Acenstry	Type of Oximeter
Henry N.R. et al. [53], 2022	26,603	2110 (7.93%)	55–73	14,397/12,206	ICU patients	Ancestry: Self-identified groups	High-fidelity pulse oximeter
Okunlola O.E. et al. [52], 2022	491	108 (22.00%)	–	–	Healthy adults under hypoxia conditions	Subjective scale: dark or light	9 brands not especified
Shi C. et al. [61], 2022	6505	–	0–69	–	Critically ill children, healthy adults, ICU patients, COVID-19 patients	Fitzpatrick phototype, Munsell scale Ancestry descriptors	–
Stell D. et al. [69], 2022	50	9 (18.00%)	19–88	–	Patients	Fitzpatrick phototype	Oxywatch MD300C19, Oxywatch MD300C29, PC- 60F Contec, CMS50D, and AM801
Allado E. et al. [70], 2021	1045	–	>18	–	Pulmonary Patients	Fitzpatrick phototype	–
Al-Naji A. et al. [30], 2021	14	–	–	9/5	Healthy adults, healthy babies	Subjective scale: dark or light	Rossmax, Model SA210
Harskamp R.E. et al. [71], 2021	69	5 (7.25%)	61–73	55/14	ICU patients	Fitzpatrick phototype	Afac FS10D, Agptek FS10C, Anapulse ANP100, Cocobear, Contec CMS50D1, Hylogy MD-H37, F4PRO Mommed YM101, Pulox PO-200, Zacurate Pro 500DL
Mosooo A. et al. [72], 2021	25	–	–	–	Healthy adults	Subjective scale: dark or light	–
Philip K.E.J. et al. [73], 2021	21	–	–	–	–	–	–
Vesoulis Z. et al. [51], 2021	294	124 (42.18%)	32–35 weeks	–	infants 32–35 weeks gestation	Ancestry: Asian-British, Black-British, White-British, White-Irish, White-other	Nellcor SpO2 Module with Neonatal-Adult MAX-N adhesive SpO2 sensor
Wiles M.D. et al. [17], 2021	194	53 (27.32%)	–	140/154	COVID-19 patients	Ancestry: White, Asian, Black	–
Sjoding M.W. et al. [50], 2020	10,001	1326 (13.26%)	–	–	ICU patients	Subjective scale: dark or light	–
Poets C.F. [74], 2019	2926	14 (0.48%)	–	–	Patients and infants with hypoxemia	Munsell	Nellcor N100, Nellcor N200, Nellcor Oximax, Nellcor N-600, Criticalcare 501, Kontron 7840, Radiometer OXI3, Masimo 4.0, Masimo SET, Novametrix 520, Radiometer Tosca/Masimo, Marquette Solar 8000, Masimo 7.9.1.0, Philips M1020A, Masimo 7.9.1.0, Ohmeda Biox III, Ohmeda Biox 3700,
Alharbi S. et al. [38], 2018	15	–	20–30	–	Healthy adults	–	Optoelectronic patch sensor, and TempIR
Baek H.J. et al. [75], 2018	28	7 (25.00%)	–	–	Healthy adults	Ancestry: African American, Caucassian, Asian	Nellcor N-550
Ebmeier S.J et al. [76], 2018	394	65 (16.09%)	47–77	150/144	ICU patients	Fitzpatrick phototype	Masimo, and Philips
Sanyal S. et al. [77], 2018	25	15 (60.00%)	20–30	15/10	Healthy adults	Fitzpatrick phototype	Biosync B-50DL
Foglia E.E. et al. [48], 2017	36	14 (40.00%)	36–39 weeks	21/15	Infants with hypoxemia	Munsell	Masimo Radical 7, and Nellcor Oximax
Kumar M. et al. [78], 2015	12	4 (33.33%)	–	7/5	Healthy adults	Subjective scale: dark or light	Texas Instruments AFE4490SPO2EVM
Bensghir M. et al. [79], 2013	1	1 (100.00%)	65	0/1	with henna	–	–
Fallow B.A. et al. [80], 2013	23	10 (43.48%)	19–43	11/12	Healthy adults	Subjective scale: dark or light	–
Feiner J.R. et al. [24], 2007	36	24 (66.67%)	19–44	19/17	Healthy adults	Ancestry: African American, Hispanic Indian, Filipino, Vietnamese	Massimo Radical, Nellcor N-595, and Nonin 9700
Bickler P.E. et al. [23], 2005	21	11 (52.38%)	24–27	–	Healthy adults	Ancestry: African American, Northern European	Nellcor N-595, Oximax-A prove, Novametrix 513, and Nonin Onyx
Hameedullah M.R. et al. [81], 2002	60	60 (100.00%)	20–45	0/60	Female with henna	–	Not reported
Wouters P.F. et al. [82], 2002	—	–	–	–	ICU patients	–	–
Adler J.N. et al. [47], 1998	284	34 (11.97%)	40-80	144/140	ICU patients	Munsell scale	Nellcor D-25, and Hayward
Avant M.G. et al. [56], 1997	50	15 (30.00%)	–	–	Critically ill children	Subjective scale: dark or light	Nellcor oxiband, and Oximax Dura-Y
Bothma P.A. et al. [46], 1996	100	100 (100%)	–	–	Critically ill adults	Portable EEL reflectance spectrometer	Simed S100, Nihon Koden, and Ohmeda 3740
Gaskin L. et al. [83], 1995	451	–	–	–	Patients, healthy adults, athletes	–	Biox II, Biox 3700, Nellcor N200, Invivo 4500, Criticalcare 501, Ohmeda 3700, Novametrix 505, Datex, Kontron 7849, Helllige 4500, Nelccor N100, Minolta Pulse Ox7
Al-Majed S.A. et al. [84], 1994	50	10 (20.00%)	–	–	Female with henna	–	–
Lee K.H. et al. [85], 1993	33	5 (15.15%)	27–92	–	ICU patients	Ancestry: Chinesse, Malay, Indian	Nellcor, Simed, and Critikon
Ralston A.C. et al. [86], 1991	–	–	–	–	–	–	–
Zeballos R.J. et al. [87], 1991	33	33 (100.00%)	–	33/0	Healthy adults	Subjective scale: dark	HP 47201A, and Ohmeda Biox IIA
Jubran A. et al. [88], 1990	54	29 (53.70%)	17–88	24/30	ICU patients	Subjective scale: dark or light	Nellcor, and Ohmeda Biox3700
Ries A.L. et al. [89], 1989	187	67 (35.83%)	–	–	Pulmonary patients	Munsell scale	Ohmeda Biox III, and HP 47201A
Mendelson Y. et al. [21], 1988	7	–	21–29	5/2	Healthy adults	Ancestry: Caucassian	HP 47201 ear oximeter, and Nellcor
Cecil W.T. et al. [43], 1988	152	16 (10.53%)	46–82	82/70	Patients	Subjective scale: dark or light	Nellcor N-100, Ohmeda 3700
Gabrielczyk M.R. et al. [45], 1988	21	4 (19.05%)	47-68	–	Patients	Subjective scale: dark or light	Nellcor N-100
Emery J.R. et al. [42], 1987	–	–	–	–	Premature Infants	Subjective scale: dark or light	–
Wang Y.T. et al. [44], 1985	31	31 (100.00%)	–	–	Patients	Subjective scale: dark or light	Ohmeda Biox III
Saunders N.A. et al. [41], 1976	52	5 (9.62%)	–	–	Healthy adults	Ancestry: African	Waters XP-350, Waters 0-lJOO, HP 47201A

### 3.1. Characteristics of the Selected Studies

Table 4 summarizes the information extracted from the included studies. Since not all the studies report the selected characteristics we indicate the number of studies that show such information between brackets. The percentage is calculated by the total number of studies that report such information.

### 3.2. Assessment of Risk of Bias

**Figure 2:**
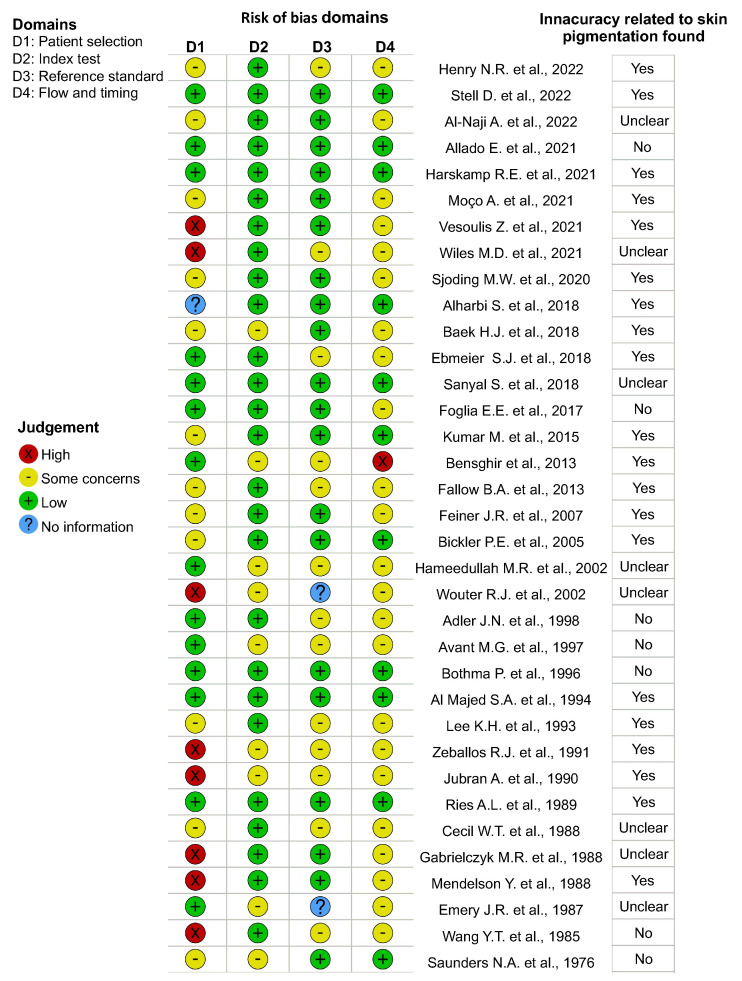
Risk of bias assessment in individual studies judged according QUADAS−2. The right column shows the qualitative final conclusion reported by the selected studies [17,21,23,30,38,41,42,43,44,45,46,47,48,50,51,53,56,69,70,71,72,75,76,77,78,79,80,81,82,84,87,88,89,93] regarding the existence of inaccuracy related to skin pigmentation.

Although there is no a standard risk of bias tool for methods-comparison studies, we follow the QUADAS-2 protocol based on four domains: patient selection, index test, reference standard, and flow and timing of patients [90]. We have considered 35/41 studies to perform risk of bias assessment of the individual studies shown in Figure 2. We exclude the reviews, letters, and the studies that did not report the clinical or experimental procedure or the data required to answer the signaling questions to assist in judgments about the risk specified in the QUADAS-2 study checklist [91]. First, one author (AMC or MFG) independently assessed the risk of bias for the selected references. Second, another author (PM, KL or DL) checked the assessment. In case of discrepancies between criteria we resolved via discussion. Last, we used the tool robvis for visualizing the risk of bias assessment [92].

We found 9/35 studies (25.71%) to be at high risk of bias for one domain [17,21,44,45,51,79,82,87,88]. The main reason for this bias is attributed to the selection of patients, where the skin pigmentation levels were classified by an unstandardized or qualitative judgment such as “dark”, “black”, “light” or “white”. Additionally, 21/35 studies (60.00%) were classified to be at unclear risk of bias for at least two domains [17,24,30,41,42,43,44,47,50,53,56,72,75,76,79,80,81,82,87,88,93]. The major issues about these domains were related to possible bias about the interpretation or conduction of the reference standard, and about flow and timing in terms of patient flow and all patients receiving the same reference standard. We also found that 6 of these studies were in both groups (at high risk of bias for one domain and unclear risk of bias for at least two domains) [17,44,79,82,87,88]. Finally, 11/35 (31.43%) studies were found to be at low risk of bias for at least three domains [23,38,46,48,69,70,71,77,78,84,89].

Furthermore, we have also included a column at the right part of Figure 2 showing the qualitative final conclusion reported by the selected studies regarding the existence of inaccuracies related to skin pigmentation. The studies are ordered by year of publication. In recent years, we can see that most of the selected studies reported inaccuracies related to the skin pigmentation. Indeed, from the 11/35 studies classified as being at low risk of bias 8/11 (72.73%) reported inaccuracies related to skin pigmentation, 3/11 (27.27%) reported no demonstrable loss of accuracy caused by skin pigmentation [46,48,70], and 1/12 (8.33%) assessed that further studies with other clinical and patient-reported data are required to understand the effects of skin pigmentation on the accuracy [77].

## 4. Discussion

### 4.1. Rq1: What Are the Most Significant Publications and the Ongoing Research Trends for Prospect Analysis on This Topic?

We have analyzed the most influential references and the progression of available publications to unravel this query. Table 5 presents the top 10 cited references per author and year, comparing total global citations (TC) from the whole bibliographic database, TC per year, local citations (LC), which are the citations received by a document by other documents included in our selection, and the percentage TC/LC, which gives a ratio between the LC internally in our selection, and global TC of a document in the whole bibliographic database. The three most influential papers were M. Kumar et al. [78] with 236 TC and an average of 29.50 cites per year, Y. Mendelson et al. [21] with 181 TC and an average of 5.17 cites per year, and A.C. Ralston et al. [86] with 149 TC and an average of 4.66 cites per year. However, the most cited reference within our selection was P.E. Bickler et al. [23] with 10 LC and a LC/TC ratio of 7.63%.

M. Kumar et al. detected inaccuracies on camera-based vital sign monitoring in dark-skinned subjects and under low lightning conditions [78]. They proposed a significant improvement in the accuracy estimation by developing a distance PPG method with a considerable reduction of the errors by comparing the algorithm on people with different skin pigmentation classified as pale/white to brown/dark. Because of the recent development of non-contact methods such as remote photoplethysmography (rPPG) or image photoplethysmography (iPPG) [94,95], this study paved the way for the advancement of new contact-less and safe methods for vital sign monitoring. Although, some methods such as iPPG are susceptible to artifacts such as surface reflections, motion artifacts, shadowing, or skin tone [96] a computer vision-based oximeter using a digital camera to measure $S_{p}O_{2}$ could be convenient, since it is a contact-less, secure and inexpensive method.

Mendelson et al. in 1988 studied different factors to consider in the design of a skin reflectance sensor for non-invasive $Sp0_{2}$ measurements [21]. The separation between the source and the detector or the skin heating effect can improve the photoplethysmographic waveforms. Indeed, the larger the distance between the photodiode and the LED’s, the larger the photoplethysmogram detected. They also found that skin heating can increase the pulse amplitude of the red and infrared photoplethysmograms. Besides, they asserted that skin pigmentation does not seem to affect the device’s accuracy. However, they performed the study in a light-skinned small population (seven Caucasian volunteers), which is not a representative sample with a different skin pigmentation classification. Besides, the review of A.C. Ralston et al. in 1991 reported a slight decrease in accuracy related to dark skin pigmentation [86]. The most cited reference in our selection by P.E. Bickler et al. also came to the same conclusion finding overestimations in a study in 3 different oximeters with a more diverse population of 21 subjects (11 dark-skinned and 10 light-skinned) [23].

**Figure 3:**
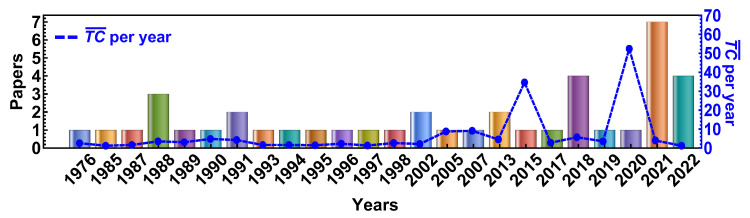
Annual publication trend of selected documents retrieved from Scopus, Pubmed and WOS between 1976 and 2022. A blue dashed line denotes the average $\bar{TC}$ per year.

Figure 3 illustrates the progression of publications available in the selected databases in the period 1976–2022. The dashed blue line indicates the yearly average total citations, while the color bars represent a document’s global citations in the whole bibliographic database. The average $\bar{TC}$ per year corresponds to the yearly average number of times each document has been cited ($\bar{TC}$ = TC/(2022-Publication Year + 1)). Two prominent peaks in 2015 and 2020 are observed, corresponding to the references of M. Kumar et al. [78] and M.W. Sjoding et al. [50] with 29.50 and 37.00 $\bar{TC}$ per year, respectively. Let us remark that the reference by M.W. Sjoding et al. has recently attracted much attention with 111 TC in the last two years. They conducted this study in two large cohorts at the University of Michigan Hospital comparing measurements of $SpO_{2}$ and arterial blood gas of a sample of over 10,000 people with 14–20% of dark-skinned patients in both cohorts. An important finding of this study is that the probability of dark-skinned patients with $SpO_{2}$ reading between 92% and 96% having an arterial oxygen saturation of less than 88% was 3 times higher (11.7%) than light-skinned patients (3.6%) with the same $SpO_{2}$ measurement. Besides, they found overestimations in dark-skinned patients with an increased risk of occult hypoxemia [50]. There was a considerable upsurge of publications in 2021. The main reason for this surge may be attributed to the increase of this technique for triage in patients because of the current COVID-19 pandemic [12,13,14,15,16,17], and also because of the recent concern about the accuracy of these devices across all skin types [20,57,58,59,60,61].

**Figure 4:**
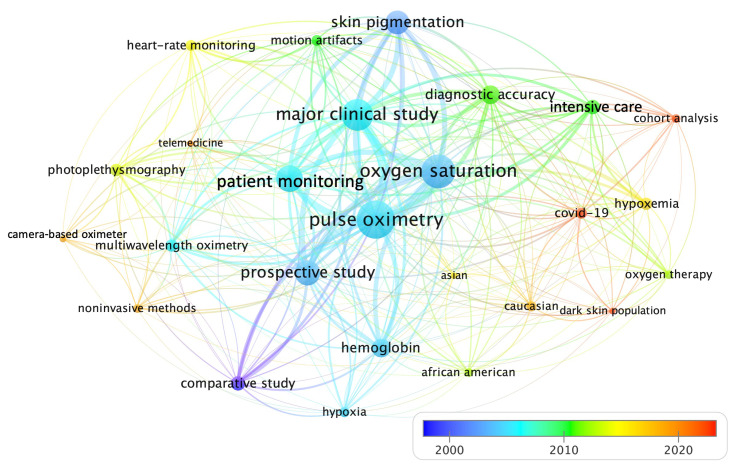
Network map of average co-occurrence citation of the most relevant keywords and dynamic view over time.

A further insight into the trending topics in terms of keywords co-occurrences is presented in Figure 4. Author’s keywords are usually associated with the publication content [97]. The total number of keywords was 413, which were too many to fit on a chart. Hence, we configured a word minimum frequency of 4, while the time-span was set at 1976 to 2022. However, to analyze the current trending topics over the last years, we just show the keywords from 2002 to 2022. We also used a thesaurus file to group the related keywords, which gives us the 30 most cited terms related to the topic. A circle represents each keyword, the larger the circle, the more a keyword has been co-cited in our selection of publications. The distance between the circles denotes the proximity of the keywords. The lines describe the co-occurrence links between two keywords. The thicker the line, the more often two keywords are mentioned together [98]. The keyword “pulse oximetry” has the strongest strength in the middle of the chart connected with the rest of the keywords as “oxygen saturation”, and “patient monitoring”. The bottom right panel is a color bar showing the dynamics of the author’s keywords. The first studies in skin pigmentation and oximetry are denoted by blue colors around 2000. The orange-red colors indicate the most recent publications.

The keyword “COVID-19” appears in 2020 near to the keywords, “hypoxemia”, “oxygen-therapy”, “intensive-care”, “diagnostic accuracy”, and “dark-skin population” among others. We found that 6 of the 41 references studied in this review examined the performance of pulse oximetry during the COVID-19 pandemic [17,30,50,51,69,71], that is the 50% of the references published since 2019. Since almost 40% of patients recovering from COVID-19 have respiratory effects derived from post-residual fibrosis after lung involvement, pulmonary rehabilitation is crucial. In this framework, “telemedicine” or telehealth, defined as the provision of health care that is offered remotely through any telecommunication tool, has recently had an enormous impact on research [99]. The limitations of contact pulse oximeter devices and the need for COVID-19 patients’ triage have encouraged research about other alternatives as camera sensors [8,70,72,77,100] to predict $S_{p}O_{2}$. The latter is reflected in the keyword “camera-based oximeter” which appears before 2020. A recent study was performed on 46 subjects at center wavelengths of 840 nm (near-infrared), 675 nm (red), and 580 nm (green) to evaluate the feasibility of calibrating a camera-based $S_{p}O_{2}$ oximeter using red and green light on data under normoxic and hypoxic conditions found significant bias at lower temperatures [72]. However, they did not stratify their results according to the level of skin pigmentation, which can introduce considerable errors.

### 4.2. Rq2: How Does Skin Color Affect the Accuracy of Pulse Oximeter Devices Incorporating Photoplethysmography?

PPG pulse oximeters depend on the detection of arterial pulsation therefore to obtain a large and stable photoplethysmogram from the back-scattered light, both the blood’s optical absorption spectrum and also the opacity of the skin should be considered [21]. Melanin attenuates the wavelength of the incident light and limits the penetration to the subcutaneous tissue because is high light-absorbing [80,101]. Because dark skin contains more melanin, several studies assert that pulse oximetry may not be reliable in dark-skinned patients [21,23,24,50,51,53,69,73,75,76,78,80,87,88,89,93].

Studies on healthy adults with different skin pigmentation showed that red and green wavelengths could estimate oxygen saturation with good agreement and lower error ratio compared to the traditional pulse oximeter [30,80,102]. Indeed, Fallow et al. found a better resolution using a green-light wavelength at rest and a green or blue-light wavelength in motion across all skin types [80].

A pioneering study to compare two pulse oximeters on 152 patients showed a statistically significant loss of accuracy in $S_{p}O_{2}$ readings for dark-skinned patients for one device [43]. Although they attributed the greater accuracy in dark-skinned patients because of the subject’s wide range of pigmentation levels, they also pointed out that these differences can be related to the LED employed in each device. Another study to determine the effect of varying LED intensity on pulse oximeter accuracy showed that in low saturation conditions, a 10:1 variation in LED intensity can lead to an error of 2.5% [103].

Accuracy errors in pulse oximeter readings have been associated with $S_{p}O_{2}$ overestimations, especially at low saturation conditions [23,24,73,93]. A review of 13 models of pulse oximeters suggested that a high percentage of carboxyhemoglobin or methaemoglobin are related to failures in saturation readings and can hide hypoxemia [86]. The effect of skin pigmentation in pulse oximeter readings under hypoxia conditions was tested in 11 dark-skinned male subjects and 10 light-skinned males, who were made to breathe an air mixture (nitrogen-carbon monoxide). The three pulse oximeters used showed overestimation in dark-skinned individuals that increases linearly as $SpO_{2}$ levels decreases [23].

A diagnostic accuracy study under the Standards for Reporting of Diagnostic Accuracy Studies [104] evaluated 10 of the most purchased pulse oximeters with arterial blood gas measurement $SaO_{2}$ as the reference standard and found a poorer $SpO_{2}$ performance in 5 of the 10 pulse oximeters. They advise that darker skin pigmentation affects the reliability of these pulse oximeters, and when used by a patient for home monitoring, confirmation of a medical-grade oximeter is required [71].

Feiner et al. conclude that pulse oximeter inaccuracy is reasonably small at $S_{p}O_{2}\;>\;80\%$ because of dark skin pigmentation [24]. However, a bias of up to 8% was detected at lower saturation conditions in dark-skinned individuals. Lee et al. also observed this bias in an older study, finding that the estimation of arterial oxygen pressure, $SaO_{2}$, varied significantly (*p* < 0.05) in multi-ethnic individuals (22 Chinese, 6 Malay and 5 Indian). The results suggested that the difference between $S_{p}O_{2}$ and $SaO_{2}$ appeared to increase with darker skin pigmentation, with overestimations more pronounced under conditions of hypoxia and jaundice [85].

Accuracy improvements were found with a crosstalk-free sensor designed by Baek et al. who performed desaturation experiments at 60% to 100% on healthy adults with different skin pigmentation [75]. The conventional sensors showed a large error in dark-skinned subjects, while the sensor which prevented optical crosstalk did not present $S_{p}O_{2}$ measurement errors according to skin color.

A prevalence of occult hypoxemia in dark-skinned subjects has been recently confirmed in three studies, showing that dark-skinned patients may have over three times the risk of experiencing occult hypoxemia during hospitalization compared to light-skinned patients [53,54,55]. This finding is of particular importance since patients with hidden hypoxemia have higher rates of in-hospital mortality and organ dysfunction [55]. A study about applying a pulse oximeter device in the titration of fractional inspired $O_{2}$ concentration in ventilator-dependent patients revealed that a $SpO_{2}$ reading of 92% was reliable when titrating supplemental $O_{2}$ in light-skinned patients receiving mechanical ventilation. However, in dark-skinned patients, the same $SpO_{2}$ value was commonly related to significant hypoxemia, and a higher $SpO_{2}$ measurements of 95%, was needed to provide a tolerable level of oxygenation [88].

The skin pigmentation effect was also tested during exercise [83,87]. A study of the reliability of two ear oximeters under normoxic and hypoxic conditions in 33 healthy male subjects (mostly with dark skin pigmentation) showed unacceptable readings for $S_{p}O_{2}$ values of less than 85% for one device and less than 90% for the other [87]. Although during exercise, the main potential errors are due to motion artifact, which complicates the use of the pulse oximeter and the accurate interpretation of the data [29,30,31], overestimations were attributed to high levels of carboxyhaemoglobin [83].

To sum up, we conclude that pulse oximetry performance in the dark-skinned population may lead to overestimations with increased incidence of occult hypoxemia and risk of negative health effects from diseases like COVID-19 [51,53,55].

### 4.3. Rq3: On Which Human Populations Have Studies Been Conducted to Verify These Discrepancies and What Methods Have Been Employed to Classify Skin Pigmentation?

Table 4 summarizes the five methods employed to classify the population studied in our selection of studies. We find that objective scales such as the Fitzpatrick Skin Pigmentation (FSP) scale or the Munsell scale were used by 6/37 studies (16.21%) [61,69,70,71,76,77] and 4/37 studies (10.81%) [47,48,74,89], respectively. Besides, a portable EEL reflectance spectrophotometer to measure natural pigmentation (not darkened by sun-tanning) was employed in 1 study ( 2.70%) [46]. On the other hand, ancestry descriptors were used in 11 studies (29.73%) [17,21,23,24,41,51,53,61,75,82,93], and other subjective scales were employed in 13 studies (35.14%) [30,42,43,44,45,50,52,56,72,78,80,87,88].

The FSP scale is commonly used to describe skin pigmentation according to the skin tanning response to UVR [105,106,107]. This scale is divided into six phototypes (SPT) from the lightest (tanning-resistant) phototype-I (SPT-I) to the darkest phototype-VI (SPT-VI). Fallow et al. classified 23 healthy subjects (11 males and 12 females, 20–59 years old) finding that phototype-V (dark brown) skin type has a significant signal-to-noise ratio than all other skin types [80]. The FSP scale was also employed to classify 404 intensive care unit patients as light (phototype I or II), medium (phototype III or IV), or dark (phototype V or VI) [76]. A small but statistically significant difference between $SaO_{2}$ and $SpO_{2}$ in light and dark skin phototypes [76] was found. In the study of the 10 most purchased pulse oximeters, 5 of 35 patients (14.3%) had a dark phototype (IV–VI) showing less accurate $SpO_{2}$ measurements [104].

In contrast, a study conducted in 298 patients classified according to the Munsell color system [108] as light group (51%), intermediate group (37%), and dark group (12%) conclude that skin pigmentation does not affect the bias or precision of pulse oximetry. They showed sub-optimal pulse oximeter function more frequently among dark-skinned patients but attributed this result to the observer bias. However, they also recognize that the study has some limitations as interrater variability or the classification by the Munsell color tile system, which requires user judgment [47]. Two more old studies, one in 1997 with a sample of 50 children, 15 of whom were dark-skinned [56] and another in 1996 with 100 dark-skinned patients [46], did not find any difference in accuracy between dark and light-skinned subjects. These studies may be misleading because of a weak correlation with skin pigmentation by using uncertain descriptors of ethnicity to classify the population as “Black” or “White”. Besides, these pulse oximeters have probably been calibrated on a population sample with light-skin pigmentation, which miss relevant interaction effects of confounding variables and skin pigmentation [23,33,109].

A multi-ethnic population study composed of 22 Chinese, 6 Malay and 5 Indian patients was also studied, finding that overestimations were more pronounced in hypoxic conditions, jaundice and darker-skinned patients [85]. The same feature was observed in the data presented in the study by Zeballos and Weisman in 33 healthy, young, nonsmoking males [87]. Although they did not refer to a specific scale of skin pigmentation degree, they also found overestimations in the two compared oximeters in darkly pigmented subjects.

Neither, Feiner et al. referred to any pre-established scale [24]. They categorized each subject’s skin as light (Caucasian), dark (African American), or intermediate (Hispanic, Indian, Filipino, and Vietnamese). They advised that a significant bias in dark-skinned patients with saturation below 80% should be considered. A similar classification was used by Baek et al. who recruited 3 dark-skinned subjects classified as “African American”, and 9 light-skinned subjects classified as “Caucasian” or “Asian” in the comparison of two pulse oximeters, one which prevented crosstalk and a conventional one [75]. They found large errors in the dark-skinned subjects with the pulse oximeter that did not prevent crosstalk.

Even the most cited references by Bickler et al. did not refer to any scale and classified the population using the ancestry “African-American” as dark-skinned and “Northern European” as light-skinned [23]. Another more recent study of 26,603 patients across four self-identified racial groups as: “White”, “Black” (i.e., “African”, “African American”, “Black”), “Asian” (i.e., “Asian”, “Indian”, “Cambodian”, “Chinese”, “Filipino”, “Japanese”, “Korean”, “Laotian”, “Pakistani”, “Taiwanese”, “Thai”, “Vietnamese”, and “American Indian” (includes “Alaskan Natives”), also found that the pulse oximetry accuracy for the detection of occult hypoxemia is not consistent across these self-identified ethnic groups with $SpO_{2}$ measurements on average overestimating arterial blood gas-derived oxygen saturation by 1.57% [53]. Besides, a subjective assessment of skin pigmentation was also employed using a scale of I to III to classify the populations as light, medium, and dark pigment levels, respectively, [43].

Female volunteers who had henna on their hands or feet were also tested [79,81,84]. They reported that black henna produces more errors in $S_{p}O_{2}$ readings than red henna [84], which is expectable since black henna produces a darker skin. These studies suggest that an alternative site (as ear oximetry) should be chosen to monitor arterial oxygen saturation [81].

Pulse oximetry is also the preferred method of $S_{p}O_{2}$ monitoring in examining every newborn and infant [110,111]. Therefore, some studies have also investigated if there is a skin pigmentation disparity in accuracy in neonatology [48,51,74]. Although one study asserted that there is no significant difference in systematic bias based on skin pigment for pulse oximetry [48], this study was not statistically significant since it had a small sample size and a stringent inclusion criterion that was not typical for premature infants. The other two studies found a small but consistent skin pigmentation disparity in $S_{p}O_{2}$ measurements with values that may be falsely high in dark-skinned preterm infants [51,74].

The risks associated with significant hypoxemia have long been studied. A study performed in 1990 in ventilator-dependent patients when titrating supplemental $O_{2}$ report inaccurate measurements in dark-skinned patients [88]. However, since they use a subjective assessment of the degree of skin pigmentation classified as light, moderately dark, and very dark, a bias related to the selection of patients should be considered. Besides, another study in 187 pulmonary patients undergoing a stress test reported technical problems in patients with slightly less accurate readings [89].

These results have important consequences because of the importance of pulse oximetry to monitor patients recovering from COVID-19 pneumonia since they require frequent and accurate $S_{p}O_{2}$ monitoring [14,73,112]. Stell et al. have recently performed a study to evaluate the characteristics of five portable pulse oximeters and their capability for home-use. They selected 50 patients classified according to the FSP scale from respiratory wards and an intensive care unit. They reported that skin pigmentation had a significant effect on measurement bias with substantial discrepancies in two models [69]. Besides, another study was performed in patients with acute COVID-19 pneumonia to find out a relationship between patient ancestry and the accuracy of pulse oximetry found a small bias between arterial blood gas analysis $SaO_{2}$ and $SpO_{2}$ measurements of 0.28%, −0.33% and −0.75% for 194 patients of “White” (135), “Asian” (34) and “Black” (19) ethnic origin, respectively, [17]. Negative percentage points to $SpO_{2}$ measurements are higher than $SaO_{2}$ values.

Let us remark that a more objective method to distribute the study subject population should be employed in the out-coming studies. Controversial and ambiguous terms such as “race” or “ethnicity” do not accurately characterize skin tone. Several studies use the term “race” for patient self-identification as “Hispanic”, “Asian”, “American Indian”, “African American”, “Malay”, “Native Hawaiian”, “Pacific Islander” or even “White”, “Black”. This categorization is as broad as inaccurate, and does not account for an accurate description of the entire spectrum of skin pigmentation.

## 5. Final Remarks

New non-invasive methods have been developed for accurate monitoring and evaluation of health variables. Pulse oximetry is a simple and low-cost and non-invasive technique for detecting oxygen saturation. However, there is growing evidence that pulse oximeters are less accurate in dark-skinned individuals at lower saturation (<80%) resulting in overestimations. Overestimation is an issue of major concern since patients may seem healthier than they are with the corresponding risk of adverse health effects from diseases like COVID-19.

Given the central role of pulse oximetry in the management of COVID-19, the reliance on pulse oximetry to triage patients in communities with dark-skinned population requires integrating of both strategic and practical recommendations to the current regulations. Biomedical sensors for vital signs monitoring should be feasible across all skin pigmentation.

Although some old studies did not find any bias related to dark skin pigmentation, the latter studies have shown that pulse oximeters devices have some limitations with dark-skinned subjects, especially at low saturation or hypoxemia conditions. Since occult hypoxemia is associated with increased mortality risk, there is a need to determine such errors exactly.

It is also worthy to remark that, to date, the effects of skin pigment on pulse oximeter performance have been studied for only a relatively small number of pulse oximeter models, and most of them have been calibrated using light-skinned individuals. To obtain reliable data to overpass the lack of features of the commercial oximeters on the market regarding dark skin pigmentation, more studies with other clinical and patient-reported data are required, and if possible, to redesign the device algorithm related to the light absorption with a calibration based on dark-skinned patients.

Furthermore, a more accurate method for classifying the research subjects into categories by degree of skin pigmentation should be employed in these studies. Using uncertain descriptors of ancestry, ethnicity or even race to define patients with dark skin pigmentation is ambiguous and also troubled on many levels. Therefore, scale such as the Fitzpatrick Skin Pigmentation or other standardized and less subjective methods employed by the dermatology community can better describe the spectrum of skin pigmentation. This would decrease the probability of inappropriate escalations of the dark-skinned population.

A holistic understanding of these results may help to develop new applied works that can support new sensor design or healthcare decision-making to avoid any potential health risk between different skin pigmentation population. However, further statistical analysis regarding how the accuracy of pulse oximetry is affected by dark skin pigmentation with a proper classification should be performed.

## Figures and Tables

**Table 1:** Search strings, keywords and items per database.

Indexing Terms	Items (n)
Web of Science	
#1 “oximet*” OR “oxygen saturation”	68,211
#2 “skin*” OR “pigmentation” OR “racial” OR “race” OR “ethnic”	2,277,504
#3 “photoplethysmography” OR PPG	7838
#4 “accuracy” OR “precision” OR “error” OR “reliability” OR “bias”	6,062,294
#5 #1 AND #2 AND #3 AND #4	74
PubMed	
#1 “oximetry” [MeSH Terms]	16,178
#2 “oximet*” OR “oxygen saturation”	44,457
#3 “skin*” OR “pigmentation” OR “racial” OR “race” OR “ethnic”	1,148,844
#4 “photoplethysmography” [MeSH Terms]	2422
#5 “photoplethysmography” OR “PPG”	7241
#6 ”accuracy“ OR ”precision“ OR ”error” OR “reliability” OR “bias”	1,835,255
#7 #2 AND #3 AND #5 AND #6	24
Scopus	
#1 “oximet*” OR “oxygen saturation”	99,915
#2 “skin*” OR “pigmentation” OR “racial” OR “race” OR “ethnic”	2,038,888
#3 “photoplethysmography” OR “PPG”	14,492
#4 “accuracy” OR “precision” OR “error” OR “reliability” OR “bias”	5,911,553
#5 #1 AND #2 AND #3 AND #4	72

**Table 2:** Main information about selected articles.

Description	Results
Timespan	1976–2022
Sources (Journals, Books, etc.)	31
Documents	41
Average citations per documents	39.95
Average citations per year per doc	3.67
References	696
Articles	34
Conference papers	1
Letter	3
Review	3
Keywords	433
Authors	171
Single-authored documents	3
Documents per Author	0.23
Authors per Document	4.28
Co-Authors per Documents	4.40

**Table 4:** Summary characteristics of the selected studies.

Items	Summary Statistics (n (%))
Type of study (41 studies)	
Prospective	32 (78.05%)
Retrospective	2 (4.88%)
Case Report	1 (2.44%)
Review	3 (7.32%)
Letter	3 (7.32%)
Experimental setting (35 studies)	
Hospital	20 (57.14%)
Laboratory	15 (42.86%)
Types of participants (39 studies)	
Infants	
<32 weeks gestation	3 (7.69%)
infants with hypoxemia	1 (2.56%)
Children	
Healthy babies	1 (2.56%)
Critically ill	1 (2.56%)
Adults	
Healthy adults	13 (33.33%)
ICU Patients	7 (17.95%)
Pulmonary patients	2 (5.28%)
COVID-19 patients	1 (2.56%)
Females with henna	3 (7.69%)
Other patients	6 (15.38%)
Athletes	1 (2.56%)
Skin pigmentation classification (37 studies)	
Fitzpatrick phototype	6 (16.21%)
Munsell scale	4 (10.81%)
Ancestry	11 (29.73%)
Subjective scale	13 (35.14%)
Black or red henna	2 (5.41%)
Portable reflectance spectrophotometer	1 (2.70%)

**Table 5:** Top 10 cited articles per author and year. TC column shows the total global citations, $\bar{TC}$ per year are the yearly average total citation, LC are local citations, while LC/TC% are the percentage ratio between local citations and global citations.

Paper	TC	$\bar{TC}$ per Year	LC	LC/TC%
Kumar M. et al., 2015 [78]	236	29.5	1	0.42
Mendelson Y. et al., 1988 [21]	181	5.17	1	0.55
Ralston A.C. et al., 1991 [86]	149	4.66	1	0.67
Bickler P.E. et al., 2005 [23]	131	7.28	10	7.63
Feiner J.R. et al., 2007 [24]	124	7.75	6	4.84
Jubran A. et al., 1990 [88]	117	3.55	5	4.84
Sjoding M.W. et al., 2020 [50]	111	37.00	6	5.41
Saunders N.A. et al., 1976 [41]	63	1.34	0	0.0
Ries A.L. et al., 1989 [89]	61	1.79	6	9.84
Fallow B.A. et al., 2013 [80]	58	5.80	0	0.0

## Data Availability

Not applicable.

## References

[1] Allen J. (2007). Photoplethysmography and its application in clinical physiological measurement. Physiol. Meas..

[2] Alian A.A., Shelley K.H. (2014). Best Practice & Research Clinical Anaesthesiology. Photoplethysmography.

[3] Nasseri N., Kleiser S., Wolf U., Wolf M. (2018). Tissue oximetry by diffusive reflective visible light spectroscopy: Comparison of algorithms and their robustness. J. Biophoton..

[4] Nitzan M., Romem A., Koppel R. (2014). Pulse oximetry: Fundamentals and technology update. Med. Devices Evid. Res..

[5] Jubran A. (2015). Pulse oximetry. Crit. Care.

[6] Martín-Escudero P., Cabanas A.M., Fuentes-Ferrer M., Galindo-Canales M. (2021). Oxygen Saturation Behavior by Pulse Oximetry in Female Athletes: Breaking Myths. Biosensors.

[7] Lee I., Park N., Lee H., Hwang C., Kim J.H., Park S. (2021). Systematic Review on Human Skin-Compatible Wearable Photoplethysmography Sensors. Appl. Sci..

[8] Kim N.H., Yu S.G., Kim S.E., Lee E.C. (2021). Non-Contact Oxygen Saturation Measurement Using YCgCr Color Space with an RGB Camera. Sensors.

[9] Martín-Escudero P. (2003). La oximetría en Registro Continuo en el Esfuerzo máximo en Distintas Especialidades Deportivas. Ph.D. Thesis.

[10] Collins J.A., Rudenski A., Gibson J., Howard L., O’Driscoll R. (2015). Relating oxygen partial pressure, saturation and content: The haemoglobin’oxygen dissociation curve. Breathe.

[11] Breuer H.W., Groeben H., Schöndeling H., Worth H. (1990). Comparative analysis of arterial oxygen saturations during exercise by pulse oximetry, photometric measurements, and calculation procedures. Int. J. Sport. Med..

[12] Scherrenberg M., Wilhelm M., Hansen D., Völler H., Cornelissen V., Frederix I., Kemps H., Dendale P. (2020). The future is now: A call for action for cardiac telerehabilitation in the COVID-19 pandemic from the secondary prevention and rehabilitation section of the European Association of Preventive Cardiology. Eur. J. Prev. Cardiol..

[13] Channa A., Popescu N., Skibinska J., Burget R. (2021). The Rise of Wearable Devices during the COVID-19 Pandemic: A Systematic Review. Sensors.

[14] Philip K.E.J., Bennett B., Fuller S., Lonergan B., McFadyen C., Burns J., Tidswell R., Vlachou A. (2020). Working accuracy of pulse oximetry in COVID-19 patients stepping down from intensive care: A clinical evaluation. BMJ Open Respir. Res..

[15] Michard F., Shelley K., L’Her E. (2021). COVID-19: Pulse oximeters in the spotlight. J. Clin. Monit. Comput..

[16] England NHS (2020). Specialty Guides for Patient Management during the Coronavirus Pandemic. Guidance for the Role and Use of Non-Invasive Respiratory Support in Adult Patients with COVID-19 (Confirmed or Suspected) 6 April 2020, Version 2. https://amhp.org.uk/app/uploads/2020/03/Guidance-Respiratory-Support.pdf.

[17] Wiles M.D., El-Nayal A., Elton G., Malaj M., Winterbottom J., Gillies C., Moppett I.K., Bauchmuller K. (2021). The effect of patient ethnicity on the accuracy of peripheral pulse oximetry in patients with COVID-19 pneumonitis: A single-centre, retrospective analysis. Anaesthesia.

[18] Shah S., Majmudar K., Stein A., Gupta N., Suppes S., Karamanis M., Capannari J., Sethi S., Patte C. (2020). Novel Use of Home Pulse Oximetry Monitoring in COVID-19 Patients Discharged From the Emergency Department Identifies Need for Hospitalization. Acad. Emerg. Med..

[19] Nematswerani N., Collie S., Chen T., Cohen M., Champion J., Feldman C., Richards G.A. (2021). The impact of routine pulse oximetry use on outcomes in COVID-19-infected patients at increased risk of severe disease: A retrospective cohort analysis. S. Afr. Med. J. Suid-Afr. Tydskr. Vir Geneeskd..

[20] World Health Organization (2021). COVID-19 Clinical Management: Living Guidance, 25 January 2021.

[21] Mendelson Y., Ochs B.D. (1988). Noninvasive pulse oximetry utilizing skin reflectance photoplethysmography. IEEE Trans. Biomed. Eng..

[22] Abay T.Y., Kyriacou P.A. (2015). Reflectance Photoplethysmography as Noninvasive Monitoring of Tissue Blood Perfusion. IEEE Trans. Biomed. Eng..

[23] Bickler P.E., Feiner J.R., Severinghaus J.W. (2005). Effects of skin pigmentation on pulse oximeter accuracy at low saturation. Anesthesiology.

[24] Feiner J.R., Severinghaus J.W., Bickler P.E. (2007). Dark skin decreases the accuracy of pulse oximeters at low oxygen saturation: The effects of oximeter probe type and gender. Anesth. Analg..

[25] Sinex J.E. (1999). Pulse oximetry: Principles and limitations. Am. J. Emerg. Med..

[26] Dünnwald T., Kienast R., Niederseer D., Burtscher M. (2021). The Use of Pulse Oximetry in the Assessment of Acclimatization to High Altitude. Sensors.

[27] Tannheimer M., Lechner R. (2019). The correct measurement of oxygen saturation at high altitude. Sleep Breath. Schlaf. Atm..

[28] Sütçü Çiçek H., Gümüs S., Deniz Ö., Yildiz S., Açikel C.H., Çakir E., Tozkoparan E., Uçar E., Bilgiç H. (2011). Effect of nail polish and henna on oxygen saturation determined by pulse oximetry in healthy young adult females. Emerg. Med. J. EMJ.

[29] Tobin R.M., Pologe J.A., Batchelder P.B. (2002). A characterization of motion affecting pulse oximetry in 350 patients. Anesth. Analg..

[30] Al-Naji A., Khalid G.A., Mahdi J.F., Chahl J. (2021). Non-Contact SpO2 Prediction System Based on a Digital Camera. Appl. Sci..

[31] De Haan G., Van Leest A. (2014). Improved motion robustness of remote-PPG by using the blood volume pulse signature. Physiol. Meas..

[32] Van Gastel M., Stuijk S., De Haan G. (2015). Motion robust remote-PPG in infrared. IEEE Trans. Biomed. Eng..

[33] Colvonen P.J. (2021). Response to: Investigating sources of inaccuracy in wearable optical heart rate sensors. NPJ Digit. Med..

[34] Zonios G., Bykowski J., Kollias N. (2001). Skin melanin, hemoglobin, and light scattering properties can be quantitatively assessed in vivo using diffuse reflectance spectroscopy. J. Investig. Dermatol..

[35] Castaneda D., Esparza A., Ghamari M., Soltanpur C., Nazeran H. (2018). A review on wearable photoplethysmography sensors and their potential future applications in health care. Int. J. Biosens. Bioelectron..

[36] Yan L., Hu S., Alzahrani A., Alharbi S., Blanos P. (2017). A Multi-Wavelength Opto-Electronic Patch Sensor to Effectively Detect Physiological Changes against Human Skin Types. Biosensors.

[37] Wassenaar E.B., Van den Brand J.G. (2005). Reliability of near-infrared spectroscopy in people with dark skin pigmentation. J. Clin. Monit. Comput..

[38] Alharbi S., Hu S., Mulvaney D., Blanos P., Raghavachari R. (2018). An applicable approach for extracting human heart rate and oxygen saturation during physical movements using a multi-wavelength illumination optoelectronic sensor system. Progress in Biomedical Optics and Imaging—Proceedings of SPIE.

[39] Costa J., Vieira H., Louro P., Vieira M., Berghmans F.M.A.G. (2018). Double junction photodiodes for multiwavelength photoplethysmography. Proceedings of SPIE—The International Society for Optical Engineering.

[40] Tamura T., Maeda Y., Sekine M., Yoshida M. (2014). Wearable photoplethysmographic sensors—past and present. Electronics.

[41] Saunders N.A., Powles A.C.P., Rebuck A.S. (1976). Ear oximetry: Accuracy and tracticability in the assessment of arterial oxygenation. Am. Rev. Respir. Dis..

[42] Emery J.R. (1987). Skin pigmentation as an influence on the accuracy of pulse oximetry. J. Perinatol. Off. J. Calif. Perinat. Assoc..

[43] Cecil W.T., Thorpe K.J., Fibuch E.E., Tuohy G.F. (1988). A clinical evaluation of the accuracy of the Nellcor N-100 and Ohmeda 3700 pulse oximeters. J. Clin. Monit..

[44] Wang Y.T., Poh S.C. (1985). Noninvasive oximetry in pigmented patients. Ann. Acad. Med..

[45] Gabrielczyk M.R., Buist R.J. (1988). Pulse oximetry and postoperative hypothermia. Anaesthesia.

[46] Bothma P.A., Joynt G.M., Lipman J., Hon H., Mathala B., Scribante J., Kromberg J. (1996). Accuracy of pulse oximetry in pigmented patients. S. Afr. Med. J..

[47] Adler J.N., Hughes L.A., Vivilecchia R., Camargo C.A.J. (1998). Effect of skin pigmentation on pulse oximetry accuracy in the emergency department. Acad. Emerg. Med. Off. J. Soc. Acad. Emerg. Med..

[48] Foglia E.E., Whyte R.K., Chaudhary A., Mott A., Chen J., Propert K.J., Schmidt B. (2017). The Effect of Skin Pigmentation on the Accuracy of Pulse Oximetry in Infants with Hypoxemia. J. Pediatr..

[49] Bent B., Goldstein B.A., Kibbe W.A., Dunn J.P. (2020). Investigating sources of inaccuracy in wearable optical heart rate sensors. NPJ Digit. Med..

[50] Sjoding M.W., Dickson R.P., Iwashyna T.J., Gay S.E., Valley T.S. (2020). Racial Bias in Pulse Oximetry Measurement. N. Engl. J. Med..

[51] Vesoulis Z., Tims A., Lodhi H., Lalos N., Whitehead H. (2021). Racial discrepancy in pulse oximeter accuracy in preterm infants. J. Perinatol..

[52] Okunlola O.E., Lipnick M.S., Batchelder P.B., Bernstein M., Feiner J.R., Bickler P.E. (2022). Pulse Oximeter Performance, Racial Inequity, and the Work Ahead. Respir. Care.

[53] Henry N.R., Hanson A.C., Schulte P.J., Warner N.S., Manento M.N., Weister T.J., Warner M.A. (2022). Disparities in Hypoxemia Detection by Pulse Oximetry Across Self-Identified Racial Groups and Associations with Clinical Outcomes. Crit. Care Med..

[54] Wong A.K.I., Charpignon M., Kim H., Josef C., De Hond A.A., Fojas J.J., Tabaie A., Liu X., Mireles-Cabodevila E., Carvalho L. (2021). Analysis of Discrepancies between Pulse Oximetry and Arterial Oxygen Saturation Measurements by Race and Ethnicity and Association with Organ Dysfunction and Mortality. JAMA Netw. Open.

[55] Valbuena V.S.M., Barbaro R.P., Claar D., Valley T.S., Dickson R.P., Gay S.E., Sjoding M.W., Iwashyna T.J. (2021). Racial Bias in Pulse Oximetry Measurement Among Patients About to Undergo Extracorporeal Membrane Oxygenation in 2019–2020: A Retrospective Cohort Study. Chest.

[56] Avant M. (1997). G; Lowe N.; Torres Jr., A. Comparison of accuracy and signal consistency of two reusable pulse oximeter probes in critically ill children. Respir. Care.

[57] Warren R., Wyden C.B. (2021). 2021.01.25 Letter to FDA re Bias in Pulse Oximetery Measurements.

[58] NHS Race & Health Observatory (2021). Pulse Oximetry and Racial Bias: Recommendations for National Healthcare, Regulatory and Research Bodies.

[59] Hunasikatti M. (2021). Racial bias in accuracy of pulse oximetry and its impact on assessments of hypopnea and T90 in clinical studies. J. Clin. Sleep Med..

[60] Holder A.L., Wong A.-K.I. (2022). The Big Consequences of Small Discrepancies: Why Racial Differences in Pulse Oximetry Errors Matter. Crit. Care Med..

[61] Shi C., Goodall M., Dumville J., Hill J.E., Norman G., Hamer O., Clegg A., Watkins C.L., Georgiou G., Alexander Hodkinson A. (2022). Article The effects of skin pigmentation on the accuracy of pulse oximetry in measuring oxygen saturation: A systematic review and meta-analysis The effects of skin pigmentation on the accuracy of pulse and meta-analysis. PLoS Med..

[62] Tranfield D., Denyer D., Smart P. (2003). Towards a Methodology for Developing Evidence-Informed Management Knowledge by Means of Systematic Review* Introduction: The need for an evidence- informed approach. Br. J. Manag..

[63] Ye S., Feng S., Huang L., Bian S. (2020). Recent Progress in Wearable Biosensors: From Healthcare Monitoring to Sports Analytics. Biosensors.

[64] Pritišanac E., Urlesberger B., Schwaberger B., Pichler G. (2021). Accuracy of pulse oximetry in the presence of fetal hemoglobin—A systematic. Children.

[65] Merigó J.M., Blanco-Mesa F., Gil-Lafuente A.M., Yager R.R. (2017). Thirty Years of the International Journal of Intelligent Systems: A Bibliometric Review. Int. J. Intell. Syst..

[66] Guerrero-Gironés J., Ros-Valverde A., Pecci-Lloret M.P., Rodríguez-Lozano F.J., Pecci-Lloret M.R. (2021). Association between Pulpal-Periapical Pathology and Autoimmune Diseases: A Systematic Review. J. Clin. Med..

[67] Aria M., Cuccurullo C. (2017). bibliometrix: An R-tool for comprehensive science mapping analysis. J. Inf..

[68] Chen C. (2006). CiteSpace II: Detecting and visualizing emerging trends and transient patterns in scientific literature. J. Am. Soc. Inf. Sci. Technol..

[69] Stell D., Noble J.J., Kay R.H., Kwong M.T., Jeffryes M.J.R., Johnston L., Glover G., Akinluyi E. (2022). Exploring the impact of pulse oximeter selection within the COVID-19 home-use pulse oximetry pathways. BMJ Open Respir. Res..

[70] Allado E., Poussel M., Moussu A., Saunier V., Bernard Y., Albuisson E., Chenuel B. (2021). Innovative measurement of routine physiological variables (heart rate, respiratory rate and oxygen saturation) using a remote photoplethysmography imaging system: A prospective comparative trial protocol. BMJ Open.

[71] Harskamp R.E., Bekker L., Himmelreich J.C.L., De Clercq L., Karregat E.P.M., Sleeswijk M.E., Lucassen W.A.M. (2021). Performance of popular pulse oximeters compared with simultaneous arterial oxygen saturation or clinical-grade pulse oximetry: A cross-sectional validation study in intensive care patients. BMJ Open Respir. Res..

[72] Moço A., Verkruysse W. (2021). Pulse oximetry based on photoplethysmography imaging with red and green light: Calibratability and challenges. J. Clin. Monit. Comput..

[73] Philip K.E.J., Tidswell R., McFadyen C. (2021). Racial bias in pulse oximetry: More statistical detail may help tackle the problem. BMJ.

[74] Poets C.F. (2019). Noninvasive Monitoring and Assessment of Oxygenation in Infants. Clin. Perinatol..

[75] Baek H.J., Shin J., Cho J. (2018). The Effect of Optical Crosstalk on Accuracy of Reflectance-Type Pulse Oximeter for Mobile Healthcare. J. Healthc. Eng..

[76] Ebmeier S.J., Barker M., Bacon M., Beasley R.C., Bellomo R., Chong C.K., Eastwood G.M., Gilchrist J., Kagaya H., Pilcher J. (2018). A Two Centre Observational Study of Simultaneous Pulse Oximetry and Arterial Oxygen Saturation Recordings in Intensive Care Unit Patients. Anaesth. Intensive Care.

[77] Sanyal S., Nundy K.K. (2018). Algorithms for Monitoring Heart Rate and Respiratory Rate From the Video of a User’s Face. IEEE J. Transl. Eng. Health Med..

[78] Kumar M., Veeraraghavan A., Sabharwal A. (2015). DistancePPG: Robust non-contact vital signs monitoring using a camera. Biomed. Opt. Express.

[79] Bensghir M., Houba A., El Hila J., Ahtil R., Azendour H., Kamili N.D. (2013). Henna dye: A cause of erroneous pulse oximetry readings. Saudi J. Anaesth..

[80] Fallow B.A., Tarumi T., Tanaka H. (2013). Influence of skin type and wavelength on light wave reflectance. J. Clin. Monit. Comput..

[81] Hameedullah, Rauf M.A., Khan F.A. (2002). Henna paste and pulse oximetry: Effect of different methods of application. J. Anaesthesiol. Clin. Pharmacol..

[82] Wouters P.F., Gehring H., Meyfroidt G., Ponz L., Gil-Rodriguez J., Hornberger C., Bonk R., Frankenberger H., Benekos K., Valais J. (2002). Accuracy of pulse oximeters: The European multi-center trial. Anesth. Analg..

[83] Gaskin L., Thomas J. (1995). Pulse Oximetry and Exercise. Physiotherapy.

[84] Al-Majed S.A., Harakati M.S. (1994). The effect of henna paste on oxygen saturation reading obtained by pulse oximetry. Trop. Geogr. Med..

[85] Lee K.H., Hui K.P., Tan W.C., Lim T.K. (1993). Factors influencing pulse oximetry as compared to functional arterial saturation in multi-ethnic Singapore. Singap. Med. J..

[86] Ralston A.C., Webb R.K., Runciman W.B. (1991). Potential errors in pulse oximetry. I. Pulse oximeter evaluation. Anaesthesia.

[87] Zeballos R.J., Weisman I.M. (1991). Reliability of noninvasive oximetry in black subjects during exercise and hypoxia. Am. Rev. Respir. Dis..

[88] Jubran A., Tobin M. (1990). Reliability of pulse oximetry in titrating supplemental oxygen therapy in ventilator-dependent patients. Chest.

[89] Ries A.L., Prewitt L.M., Johnson J.J. (1989). Skin color and ear oximetry. Chest.

[90] Whiting P.F., Rutjes A.W.S., Westwood M.E., Mallett S., Deeks J.J., Reitsma J.B., Leeflang M.M.G., Sterne J.A.C., Bossuyt P.M.M. (2011). QUADAS-2: A revised tool for the quality assessment of diagnostic accuracy studies. Ann. Intern. Med..

[91] Whiting D.P. Risk of Bias and Applicability Judgments. https://www.bristol.ac.uk/population-health-sciences/projects/quadas/quadas-2/.

[92] McGuinness L.A., Higgins J.P.T. (2020). Risk-of-bias VISualization (robvis): An R package and Shiny web app for visualizing risk-of-bias assessments. Res. Synth. Methods.

[93] Lee T.Q., Barnett S.L., Shanfield S.L., Anzel S.H. (1990). Potential application of photoplethysmography technique in evaluating microcirculatory status of STAMP patients: Preliminary report. J. Rehabil. Res. Dev..

[94] Khanam F.T.Z., Perera A.G., Al-Naji A., Gibson K., Chahl J. (2021). Non-Contact Automatic Vital Signs Monitoring of Infants in a Neonatal Intensive Care Unit Based on Neural Networks. J. Imaging.

[95] Liu H., Ivanov K., Wang Y., Wang L. (2015). A novel method based on two cameras for accurate estimation of arterial oxygen saturation. Biomed. Eng. Online.

[96] Guazzi A.R., Villarroel M., Jorge J., Daly J., Frise M.C., Robbins P.A., Tarassenko L. (2015). Non-contact measurement of oxygen saturation with an RGB camera. Biomed. Opt. Express.

[97] Song Y., Chen X., Hao T., Liu Z., Lan Z. (2019). Exploring two decades of research on classroom dialogue by using bibliometric analysis. Comput. Educ..

[98] van Eck N.J., Waltman L. (2010). Software survey: VOSviewer, a computer program for bibliometric mapping. Scientometrics.

[99] Wosik J., Fudim M., Cameron B., Gellad Z.F., Cho A., Phinney D., Curtis S., Roman M., Poon E.G., Ferranti J. (2020). Telehealth transformation: COVID-19 and the rise of virtual care. J. Am. Med. Inform. Assoc. Jamia.

[100] van Gastel M., Stuijk S., de Haan G. (2016). New principle for measuring arterial blood oxygenation, enabling motion-robust remote monitoring. Sci. Rep..

[101] Fine J., Branan K.L., Rodriguez A.J., Boonya-ananta T., Ajmal, Ramella-Roman J.C., McShane M.J., Coté G.L. (2021). Sources of Inaccuracy in Photoplethysmography for Continuous Cardiovascular Monitoring. Biosensors.

[102] Maeda Y., Sekine M., Tamura T. (2011). The advantages of wearable green reflected photoplethysmography. J. Med. Syst..

[103] de Kock J.P., Reynolds K.J., Tarassenko L., Moyle J.T. (1991). The effect of varying LED intensity on pulse oximeter accuracy. J. Med. Eng. Technol..

[104] Bossuyt P.M., Reitsma J.B., Bruns D.E., Gatsonis C.A., Glasziou P.P., Irwig L., Lijmer J.G., Moher D., Rennie D., de Vet H.C.W. (2015). STARD 2015: An updated list of essential items for reporting diagnostic accuracy studies. BMJ.

[105] Fitzpatrick T.B., Breathnach A.S. (1963). The epidermal melanin unit system. Dermatol. Wochenschr..

[106] Fitzpatrick T.B. (1988). The validity and practicality of sun-reactive skin types I through VI. Arch. Dermatol..

[107] Moreiras H., O’Connor C., Bell M., Tobin D.J. (2021). Visible light and human skin pigmentation: The importance of skin phototype. Exp. Dermatol..

[108] Munsell A.H. (1915). Atlas of the Munsell Color System.

[109] Xiao K., Yates J.M., Zardawi F., Sueeprasan S., Liao N., Gill L., Li C., Wuerger S. (2017). Characterising the variations in ethnic skin colours: A new calibrated data base for human skin. Skin Res. Technol..

[110] Kugelman A., Wasserman Y., Mor F., Goldinov L., Geller Y., Bader D. (2004). Reflectance Pulse Oximetry from Core Body in Neonates and Infants: Comparison to Arterial Blood Oxygen Saturation and to Transmission Pulse Oximetry. J. Perinatol..

[111] Hay W.W.J., Brockway J.M., Eyzaguirre M. (1989). Neonatal pulse oximetry: Accuracy and reliability. Pediatrics.

[112] Luks A.M., Swenson E.R. (2020). Pulse oximetry for monitoring patients with COVID-19 at home potential pitfalls and practical guidance. Ann. Am. Thorac. Soc..

